# Femtoplankton: What’s New?

**DOI:** 10.3390/v12080881

**Published:** 2020-08-12

**Authors:** Jonathan Colombet, Maxime Fuster, Hermine Billard, Télesphore Sime-Ngando

**Affiliations:** Laboratoire Microorganismes: Génome et Environnement (LMGE), UMR CNRS 6023, Université Clermont Auvergne, F-63000 Clermont-Ferrand, France; maxime.fuster@uca.fr (M.F.); hermine.billard@uca.fr (H.B.); telesphore.sime-ngando@uca.fr (T.S.-N.)

**Keywords:** femtoplankton, biomimetic mineral–organic particles, extracellular vesicles, viruses, gene transfer agents, ultra-small prokaryotes, aster-like nanoparticles

## Abstract

Since the discovery of high abundances of virus-like particles in aquatic environment, emergence of new analytical methods in microscopy and molecular biology has allowed significant advances in the characterization of the femtoplankton, i.e., floating entities filterable on a 0.2 µm pore size filter. The successive evidences in the last decade (2010–2020) of high abundances of biomimetic mineral–organic particles, extracellular vesicles, CPR/DPANN (Candidate phyla radiation/Diapherotrites, Parvarchaeota, Aenigmarchaeota, Nanoarchaeota and Nanohaloarchaeota), and very recently of aster-like nanoparticles (ALNs), show that aquatic ecosystems form a huge reservoir of unidentified and overlooked femtoplankton entities. The purpose of this review is to highlight this unsuspected diversity. Herein, we focus on the origin, composition and the ecological potentials of organic femtoplankton entities. Particular emphasis is given to the most recently discovered ALNs. All the entities described are displayed in an evolutionary context along a continuum of complexity, from minerals to cell-like living entities.

## 1. Introduction

**Figure 1:**
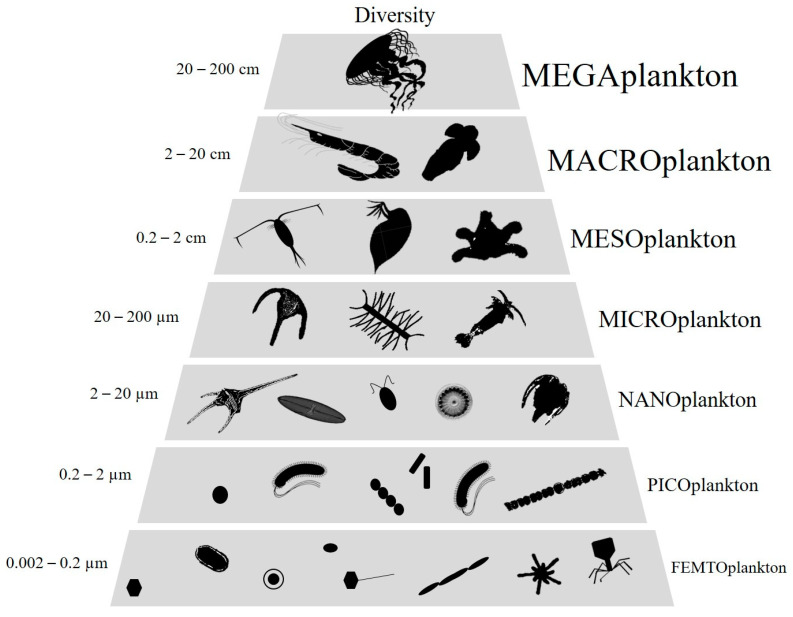
Schematic representation of plankton size classes (adapted from [2]).

Victor Hensen first introduced the term “plankton” in 1887 to define all organisms that live in suspension in water and have limited locomotion power to maintain their position against currents. Plankton was first divided into broad functional groups according to their trophic levels but practical reasons related to their study has led to a classification by size classes [1]. Thus, Sieburth classified plankton into size ranges covering eight orders of magnitude from femto- (0.02–0.2 µm) to mega-plankton (>20 cm) [2] (Figure 1). While the largest size fraction has proved to be very diversified, with the occurrence of various phylogenetic groups, the smallest one, i.e., femtoplankton, has long been considered to be exclusively composed of virus-like particles (VLPs) [2,3]. Over the last two decades, technical advances in molecular and microscopic sciences have revealed an unexpected and underestimated diversity of femtoplankton entities other than viruses, including, for example, various tiny prokaryotes CPR (Candidate phyla radiation), DPANN (Diapherotrites, Parvarchaeota, Aenigmarchaeota, Nanoarchaeota and Nanohaloarchaeota) [4] and, more recently, intriguing aster-like nanoparticles (ALNs) that we have reported in various aquatic systems [5]. These discoveries lead to a necessary reconsideration of the femtoplankton compartment in terms of diversity and associated ecological potentials. We refer here to “femtoplankton entities” as those that (i) are totally or partially organic, (ii) can be filterable on a 0.2 µm pore size filter, (iii) are bounded by an outer membrane, “membrane-like” or wall structure and (iv) have the ability to multiply or divide independently or not. Fully inorganic or non-biotic nanoparticles (from 1 to 100 nm) and molecular colloids (from 1 to 1000 nm) populating aquatic systems have been the subject of excellent reviews and will not be discussed here [6,7]. In addition, miniaturized prokaryotes and *Candidatus Pelagibacter ubique* that are on the border between femto- and nanoplankton are also excluded from this review [8,9,10,11].

This review focuses on the diversity of femtoentities in aquatic ecosystems, with an emphasis on the origin and composition of their different representatives and the associated ecological potentials. The femtoplankton entities treated in this review are presented in an evolutionary context, along a gradient of progressive increase in complexity, ranging from mineral–organic entities (biomimetic mineral–organic particles/nanobes) to fully biotic entities (VLPs—i.e., viruses, subviral, agents and gene transfer agents—extracellular vesicles and prokaryotes). Particular attention is given to the recently discovered aster-like nanoparticles [5].

## 2. From Mineral to Biotic Entities: A Path Toward the Living Being?

### 2.1. Biomimetic Mineral–Organic Particles and Nanobes

The discovery and characterization of new femtoplankton entities collides with the concept of the origin and emergence of a cell life form. Two major theories (“The RNA world” vs. “The metabolism-first”) and two approaches (“top-down biology” vs. “bottom-up chemistry”) have historically competed over the research of the starting point of life on the primitive Earth and the primordial stages of life evolution [12,13,14,15,16,17,18,19]. Many scenarios to explain the emergence of the first cell life form arise from these lines of research and from the possible location where life appeared (e.g., submarine hydrothermal vents, pumice rafts, volcanic-hosted splash pools, subaerial geysers, etc., reviewed in [20,21]). However, at present, there is no experimental evidence of a consensus scenario (discussed in [19,22,23,24] and references herein). A recurring feature in the evolutionary process that could lead to the first life form is the gradual increase in complexity from inorganic nanoparticles to the emergence of the cell. Baum [25] resumed that the cell could find its origin with the creation of chemical consortia adsorbed on mineral surfaces. The selection processes would eventually give rise to limited entities reproducing independently, such as cells.

**Figure 2:**
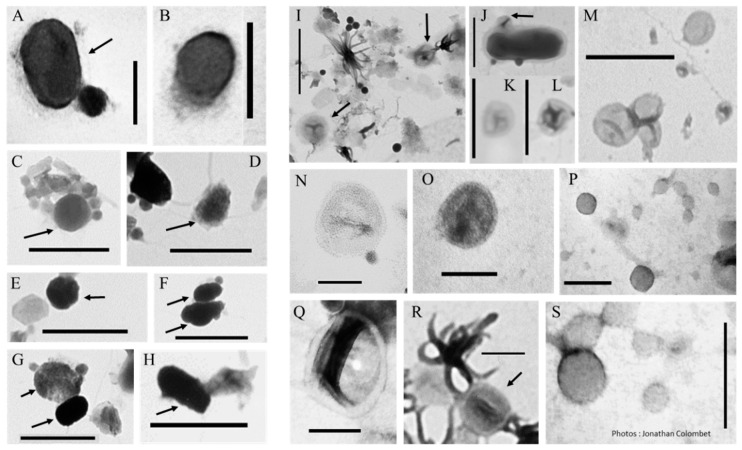
Transmission electron micrographs of biomimetic mineral–organic- (BMOPs) and/or nanobe-like particles (**A**–**H**) and vesicle-like particles (**I**–**S**). Arrows indicate the target particles when the samples are heterogeneous. (**A**,**B**,**N**–**S)** scale bar: 100 nm, (**C**–**H**,**I**–**M**) scale bar: 500 nm.

Biomimetic mineral–organic particles (BMOPs), including the majority of controversial “nanobes” also known as “nanobacteria”, “nanobacteria” or “calcifying nanoparticles” [26,27,28,29,30,31,32,33,34], could be considered as a first step in complexification leading to the genesis of a cell type structure, known as a protocell. The formation of the majority of BMOPs/nanobes could be the result of physico-chemical processes (e.g., aggregation) that are entirely abiotic, or combinations of minerals and molecules derived from biological entities [29,30,33]. Nevertheless, these entities present intriguing features. They have the potential to generate mineral–organic amalgams that are able to replicate themselves via cell-like processes, such as symmetrical fission and they have the ability to mimic various life forms (e.g., coccoid, amoeboid, ovoid, filamentous, etc.), such as microscopic fungi (Actinomycetes) and prokaryotes [26,27,35,36,37]. However, their tiny size (between 20 and 1000 nm in diameter, like those in Figure 2A–H) results, in most cases, in volumes largely under the theoretical minimal cell volume (TMCV, i.e., 0.008 µm^3^) required to house nucleic acids and the associated biosynthetic machinery required for a self-sufficient form of life [26,27,33,38]. The origin and characteristics of some of these biomimetic amalgams, mainly those known as “nanobes”, remain poorly understood.

The composition of these aggregates of minerals and organic particles is representative of the environment in which they evolved, including human and cow blood, terrestrial minerals, extraterrestrial meteorites, and aquatic environments [26,27,29,33,36,39,40,41]. Since these entities are ubiquitous, we speculate that their diversity is likely at least as great as the number of possible mineral–organic combinations in the environment and is similar or greater than the known biological diversity of the past and contemporary living world.

Carbon, oxygen, nitrogen, calcium, phosphorus, silicon, iron, sodium, magnesium, manganese, fluorine, aluminum, barium, sulfur, zinc, potassium, terbium, chlorine and cobalt are examples of elements that may be included in BMOP and nanobe composition [33,42]. This non-exhaustive list may combine mineral phases dominated by several compounds (e.g., calcium and iron sulfates, silicon and aluminum oxides, sodium carbonate, iron sulfide and hydroxides containing iron, manganese, aluminum) with organic phases of complex composition (e.g., humic materials, peptides, proteins, lipids, peptidoglycans, polysaccharides) ([33,42] and references herein). It is interesting to note that BMOPs from human samples can include a wide range of proteins with complex biological functions, such as coagulation factors, calcification inhibitors, complement proteins, protease inhibitors, or lipid carriers [43]. The presence of nucleic acids in BMOPs and nanobes remains a controversial issue. Some authors have reported positive detection of nucleic acid using various markers [27,30]. Raoult et al. [30] suggested that BMOPs and nanobes do not contain nucleic acid and that this positive detection could be the result of the labeling of contaminating nucleic acids trapped on the target particle. These results strongly suggest that environmental BMOPs and nanobes are potential carriers of genetic information and associated biological functions. It is therefore very important to strengthen the research efforts on their origin, composition, diversity, and ecological potentials through their interactions with biotic and abiotic environments.

Many authors, in agreement with the origin, composition and the theoretical formation pattern of BMOPs and nanobes, have classified them as non-living forms [29,30,33,35]. However, some controversial “nanobes” remain mysterious and further work is needed to clearly elucidate their exact nature and to understand the potential role of BMOPs and nanobes in the evolution of life [26,27,32,41]. Indeed, if they are not living entities, BMOPs and nanobes can be considered as an evolutionary step towards cell formation through the formation of mineral–organic complexes. Their composition and organization into cell type structures (including a membrane mimicking the cell wall, [27]) could be consistent with the beginning of the compartmentalization process known as one of the critical steps in the genesis of earlier free or symbiotic cell forms.

### 2.2. Extracellular Vesicles

The progressive increase in complexity of bio-mineral complexes at cell emergence requires a critical step where a boundary occurs and separates the living cell system from its environment [44,45,46]. The compartmentalization of the primordial soup (i.e., the process that allowed the isolation and creation of a physico-chemical and thermodynamic environment suitable for the synthesis of bio-macromolecules) into vesicles would have favored the emergence of primitive life forms [47]. These authors also suggested that membrane vesicles could have a role in early cell evolution and may have helped shape the nature of LUCA, our Last Universal Common Ancestor. Thus, lipid vesicles may have been the first protocells to concentrate RNA before the appearance of ribocells, ancestors of RNA-based cells, that preceded LUCA [47]. Beside this evolutionary theory, there is no doubt that extracellular vesicles (EVs) represent the smallest cell-like entities surrounded by a lipid structure.

The biology of EVs has been widely documented over the last decade, mainly in terms of their origin, composition, diversity and biological purposes (see references in [48,49]). Scientists reported that EV production is a universal and conserved process that occurs in all branches of the tree of life: bacteria, archaea and eukaryotes [50,51]. EVs are diverse in origin and composition, and there is little consensus on their classification [52]. Bacterial vesicles are represented by extracellular vesicles (20–250 nm) or outer membrane vesicles (20–230 nm) while archaea produce membrane vesicles (50–230 nm). Eukaryotic EVs can be grouped into three main groups: microvesicles (50–1000 nm), exosomes (30–150 nm) and apoptotic bodies (500–2000 nm) [49,53]. With the exception of apoptotic vesicles and large microvesicles, the other EVs are spherical nanoparticles [50,54,55,56,57]. These particles could therefore be found in plankton where they can be confused with other nanoparticles (Figure 2I–S). Since all living cells on earth are probably capable of producing vesicles, we assume that their diversity could be as great as that of their parent cells.

Bacterial vesicles are formed by budding of the cytoplasmic, outer and outer-inner membrane. The composition of their membrane and lumen is therefore reminiscent of the membrane, periplasm and cytoplasm of their producer cells. For example, EVs may contain soluble proteins, membrane proteins, lipoproteins, phospholipids and glycolipids from the donor cells membrane. All of these molecules are involved in essential cell membrane functions, such as substances transfer, cell adhesion, ion conductivity, cell signaling, binding surface for several extracellular structure, etc. [47,48,49,50]. They also carry elements of the cytoplasm of the producing cells, such as toxins, DNA, RNA, immunomodulatory compounds, communication factors, adhesins, adenosine triphosphate (ATP), enzymes involved in the degradation of peptidoglycans or antibiotics, virulence factors (anthrolysin, coagulases, lipase), etc. ([47,48] and references therein). The quality and quantity of molecular loads differ greatly from one EV to another. For example, in the marine environment, Biller et al. [58] demonstrated that the size and quantity of DNA varied between different bacterial taxa and that only a small proportion of EVs contain DNA.

Knowledge about the vesicles produced by archaea is less extensive and still in its infancy compared to bacteria. In aquatic environments, the models studied (mainly Sulfolobus and Thermococcales) show that archaea EVs are membrane vesicles produced by cytoplasmic membrane. The biology of these models, in terms of origin, composition, diversity and biological purpose, is detailed in [48]. As with bacteria, the composition of archaea vesicles is inherited from their producing cell. For example, membrane vesicles produced by *Sulfolobus* or *Thermococcus species* harbor S-layer proteins and the oligopeptide-binding protein OppA obtained from parental cells [59,60,61,62]. The EVs of three *Sulfolobus* species carry various proteins identified as having potential implications in cell division (ESCRT, Vps4), adhesion, migration, homing, pattern formation and signal transduction (vWA), as well as in signaling, endocytosis (flotillin), cyanure detoxification (thiosulfate sulphur transferase) and antimicrobial processes (sulfolobicin) [47,48]. EVs produced by *Thermococcus* species are often associated with genomic DNA or RNA [60,61,63]. Recently, Erdmann et al. [64] described a new type of EV containing plasmid in a psychrophilic halophilic archaea *Halorubrum lacusprofundi*.

The origin and composition of eukaryotic vesicles (i.e., exosomes and microvesicles) are well documented (see reviews in [48,49]). Gill et al. [48] mentioned that the release of EVs in the environment is characteristic, and probably conserved, in all eukaryotic cell types (i.e., animals, plants, protists and fungi), including single and multicellular organisms. As such, they may be present in all types of environments, including aquatic systems. However, most studies to date have been conducted in animals, mainly in terrestrial mammalian models such as mice and humans [49]. Exosomes are formed through the endocytic pathway from the “outward” budding of the late endosomal membrane [65,66]. They can accumulate in multivesicular bodies during the endosomal pathway and can be released into the environment after fusion with the plasma membrane [50,67,68,69,70]. Microvesicle EVs are formed from direct outward budding or pinching of the cell’s plasma membrane [71]. In some cases, they are released from tubular structures that are extensions of the plasma membrane [72,73]. Exosomes and microvesicles are formed by packaging the cytoplasmic contents in membrane-bound vesicles and have been shown to carry all types of cellular components. Extensive reviews on cargo molecules and their functions have already been provided [48,49,74]. For example, microvesicles can contain proteins involved in cell adhesion, motility, activation and proliferation (tetraspanins and associated proteins). Other cargo proteins can also be present, such as those fundamental in pathogen recognition (immunoglobulins), cytoskeletal properties (tubulin and actin), vesicular trafficking (Rab GTPase proteins, annexins, stomatin, prohibitin, flotillin) or cell division (ESCRT-related proteins), etc. In addition, many lipids, including sphingomyelin, cholesterol, ganglioside GM3, desaturated lipids, phosphatidylserine and ceramide are also intravesicular components of eukaryotic EVs, as well as genetic materials [75,76,77,78,79]. These include a large amount of mRNA and sRNA, single-stranded DNA, mitochondrial DNA, plasmid DNA and double-stranded DNA [80,81,82,83,84].

Overall, EV composition varies greatly depending on the phylogenetic position, lifestyle and physiological state of the parental cells, as well as prevailing environmental conditions (reviewed in [85]). They represent a huge reservoir of biomolecules and are essential vectors in the aquatic environment. Protein and nucleic acid contents mainly derived from parental cells but also from their viruses and other symbionts [47,48,86]. These authors suggested many potential interactions between viruses and EVs, in both evolutionary and physiological contexts. Gill and Forterre [47] proposed the existence of ribovirocells, derived from lipid vesicles, which evolved into virocells at the origin of viruses. EVs can be used as decoys against viral attack but virus-infected cells also produce EVs that enhance viral infection (reviewed in [48]). Improving our knowledge of the biology and ecology of EVs is essential for understanding the origin of viruses [87,88].

### 2.3. Viruses and Gene Transfer Agents

#### 2.3.1. Viruses

The genesis of EVs is an example of biological compartmentalization based on lipid arrangements and boundaries. Biological compartmentalization may also result from protein or protein–lipid arrangements and boundaries which are characteristics of encapsidated and enveloped viruses. The origin of viruses is widely debated. Three main hypotheses have been formulated, namely the progressive (or escape) hypothesis, the regressive (or reduction) hypothesis and the virus-first hypothesis. Krupovic and Koonin [89] defined these hypotheses as follows. The progressive hypothesis postulates that viruses evolved independently in different domains of life from cellular genes that embraced selfish replication and became infectious. The regression hypothesis submits that viruses are degenerated cells that have succumbed to obligatory intracellular parasitism and in the process, have shed many functional systems that are ubiquitous and essential in cellular life forms, particularly the translation apparatus. Finally, the virus-first hypothesis, also known as the primordial virus world hypothesis, views viruses (or virus-like genetic elements) as intermediaries between prebiotic chemical systems and cellular life and therefore postulates that virus-like entities are derived from the precellular world. These hypotheses are well discussed in [90]. In addition to the possible co-evolution between viruses and EVs [91], it is clear that the complexity of these particles increases from vesicles to viruses, to living cells. In the virus-first hypothesis, Koonin [92] suggested that capsid is a primitive form that may have paved the way for the formation of the extant complex membranes of modern cells. This author mentioned that viral particles could have served as a “laboratory” to test molecular devices that were then incorporated into the membranes of emerging cells. Gill and Forterre [47] proposed that viruses may have existed prior to the appearance of the first cell and that they could be descendants of lipid vesicles through the formation of ribovirocells, prior to the emergence of RNA virions.

**Figure 3:**
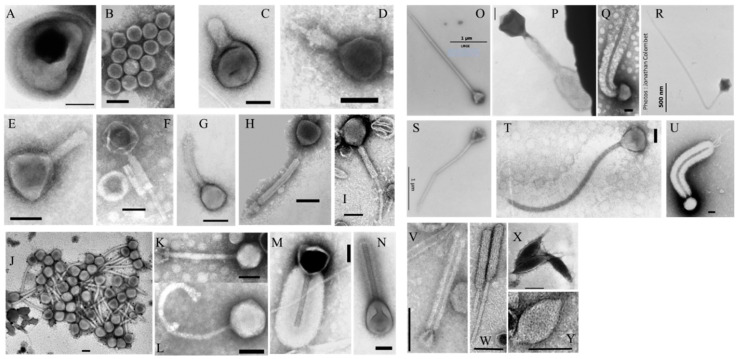
Transmission electron micrographs of different morphotypes of virus-like particles (VLPs). (**A**) VLP embedded in a vesicle-like structure. (**B**) Tailless VLP. (**C**–**N**) Tailed VLPs representing phages like morphotypes. (**O–U**) Giant tailed VLPs. (**V**–**Y**) Archaea-like viruses. Scale bars = 100 nm, excepted notifications (**O**,**S**,**R**).

Viruses are acellular biological entities (Figure 3) unable to reproduce without their cell hosts. They have a genome consisting of DNA or RNA that could be double-stranded or single-stranded, linear or circular, segmented or unsegmented. The genome is encapsulated in a protein coat called a capsid (with exception of Endornaviridae, Hypoviridae, Narnaviridae), which in specific cases can be enveloped by lipid membranes. All types of life forms, from prokaryotes (bacteria and archaea) to eukaryotes (animals and plants), can be infected by one or more viruses [93,94,95,96]. The diversity of viruses is therefore probably at least as great as that of their susceptible hosts [97]. Viruses have been classified according to a combination of different criteria: type (RNA or DNA) and form (single- or double-stranded; circular or linear) of the nucleic acids; the different ways in which they produce mRNA; morphology of viral particle; host type; and presence/absence of an envelope ([98,99] and references herein). Thus, seven groups, organized into taxonomic levels, have been delineated: (i) positive-stranded RNA viruses, (ii) negative-stranded RNA viruses, (iii) double-stranded (ds) RNA viruses, (iv) reverse-transcribing viruses with positive-stranded RNA genomes, (v) reverse-transcribing viruses with ds DNA genomes, (vi) single-stranded (ss) DNA viruses, and (vii) dsDNA viruses [100,101]. With the development of high-throughput sequencing technologies [102,103,104,105], the International Committee on Taxonomy of Viruses (ICTV) has considerably simplified the classification criteria by allowing all viruses to be classified on the basis of genome-sequence information [106]. The classification based on genome-sequence information opens up a new way in the classification of viruses that are known only from metagenomic data [97,107]. Although no universally shared sequences are conserved across the entire genome of the viral world, the genomic approach also allows to target the phylogeny of specific viruses harboring common genetic markers [108]. The taxonomic classification of viruses is constantly evolving. In March 2020, the ICTV has identified 4 realms, 9 kingdoms, 16 phyla, 2 subphyla, 36 classes, 55 orders, 8 suborders, 168 families, 103 subfamilies, 1421 genera, 68 subgenera and 6590 species [106]. Note also the existence in femtoplankton of subviral agents which are not classified in the same way as viruses [106,109,110,111]. These subviral agents are composed of three varieties: satellite viruses, viroids and prions (widely defined and described in [106,109]). They mainly infect plants, fungi, and/or vertebrates. Satellite viruses are subviral agents morphologically indistinguishable from ordinary viral particles lacking genes capable of encoding functions necessary for replication. Thus, for their multiplication, they depend on the co-infection of a host cell with a helper virus. Viroids are small, circular, single-stranded, non-protein-coding RNAs that replicate autonomously when inoculated into higher plants. Prions are infectious protein particles devoid of nucleic acids.

To date, dsDNA and ssDNA viruses dominate the viral pool of the bacterial and archaeal communities. In contrast, positive-stranded RNA and dsRNA viruses are rare in these communities, while retroviruses are absent [112]. In eukaryote communities, RNA and retroviruses are dominant, with diversity and abundances far exceeding that of DNA viruses [112,113,114]. The size of the virus genome varies by about four orders of magnitude, with the smallest (0.859 kbp) recorded in ssDNA Circovirus SFBeef and the largest (2473 kbp) in dsDNA *Pandoravirus salinus* [115]. RNA viruses have the smallest genomes compared to other viruses [115,116]. The capsid of viruses results from the arrangement of multiple copies of one or a few different proteins that determine their shape and size. Viruses harbor a remarkable variety of conformations (helical, polyhedral, spherical, ovoid, bacilliform, bullet-shape). Archaeal viruses have additional original forms (bottle-, lemon-, rod-shape). Some viruses are tailless (animal and plant viruses), while others present contractile or non-contractile tail (prokaryotic viruses = phages) characteristics of the families Ackermannviridae, Herelleviridae, Myoviridae, Siphoviridae and Podoviridae [106]. There are also viruses without a true capsid (Endornaviridae, Hypoviridae and Narnaviridae, for example); these are mostly parasites of eukaryote microorganisms or plants. The presence of an outer envelope in an “enveloped virus” combines virally encoded proteins with lipids and/or carbohydrates derived from the host cell membrane, depending on the viral family or genus [116]. Viruses vary in size and in diameter from 17 to over 400 nm for icosahedral forms, while filamentous forms vary in length from 650 to over 1950 nm [116,117]. As non-motile entities, viruses meet their hosts by diffusive transport according to fluidic dynamic concepts (Brownian movement) or via biological or inanimate vehicles. The components of the capsid, tail or viral envelope, mainly proteins, play a crucial role in the recognition and in the specific binding of viruses to host cell receptors. Several stages can be distinguished in the life cycle of a virus: adsorption, penetration of nucleic acids and uncoating, expression and replication of the nucleic acids, virion assembly and release [118]. Viral replication strategies range from obligatory host lysis (lytic cycle) to the persistence of viral genomes within hosts (lysogenic cycles), with strategies intermediate between these extremes (e.g., chronic infections) [119]. In eukaryote viruses, a remarkable feature is the high diversity of genetic cycles, depending on nucleic acid content [94,120].

Viruses are found wherever life is possible. The aquatic environment undoubtedly represents the largest reservoir of viral biodiversity on earth ([93,121,122,123,124,125,126,127,128] and associated references). In such an environment, metagenomic datasets have revealed the existence of numerous giant phages and their associated virophages [129,130]. The genomes of some giant viruses are larger than those of many bacteria and archaea [131,132,133,134,135]. Genetic repertoires include various components of the viral world that have not previously been described (CRISPR–Cas systems, transfer RNAs (tRNAs), tRNA synthetases, tRNA-modification enzymes, translation-initiation and elongation factors and ribosomal proteins) [129]. These components are associated with functions that are characteristics of cellular organisms (translation machinery, DNA maintenance, and metabolic enzymes) [136]. Al-Shayeb et al. [129] argued that the characteristics of giant viruses, distinct from those of small phages and partially analogous to those of symbiotic bacteria, blur the distinctions between life and non-life. Finally, although there is no consensus on the scenario explaining the origin of viruses and their living or non-living nature, it is now accepted that they have been involved in the genesis and/or the evolution of cellular life forms.

#### 2.3.2. Gene Transfer Agents (GTAs)

While viruses are implicated in cellular genesis and/or evolution, some of them have been suspected of drifting into gene transfer agents (GTAs), in a process that has been conceptualized as “prophage domestication” [137,138,139]. Briefly, GTAs arise though deletion and recombination processes that place the structural and DNA-packaging genes of prophage under the control of cellular regulators [140]. GTAs are suspected to be actively maintained by natural selection acting on benefits they confer [140]. GTAs are defined as tailed phage-like particles (with structural similarities to established phage morphotypes such as siphoviruses and podoviruses) that contain a random fragment of the genome of the producer cell genome [141,142]. Their capsid sizes vary from 30 to 80 nm and they contain 4–14 kbp of DNA packaged in a protein capsid shell [143,144,145,146,147,148,149]. The stages of GTA production show similarities with those of lysogenic infection from specific attachment to the release of GTAs into the extracellular environment via lysis of the producer cell [150,151,152]. However, unlike the prophage genes, the genes encoding GTAs are not excised from the genome of the host cell. GTAs are not replicative. The amount of DNA it contains is insufficient to encode the protein components of the particle itself. Therefore, a GTA particle does not necessarily contain genes encoding GTA, and cannot transfer a complete set of GTA structural genes to a recipient cell. This is distinct from a generalized transducer phage, for which usually only an occasional particle contains host genes, and the fragments of packaged DNA are the size of the phage genome [153]. GTAs have now been documented in a wide range of prokaryotes, including bacteria and archaea [144,148,149,154,155,156,157,158]. The production of GTA particles depends on the physiology of the host, and the factors regulating GTA production differ from organism to organism [159]. It is possible that GTAs exist in abundance in all Earth environments in which they act primarily as mediators of horizontal gene transfer through a mechanism similar to transduction [159]. Identifying GTAs and distinguishing them from other femtoplankton particles, especially viruses, is a challenge. Many additional details concerning GTAs are provided in previous reviews [140,141,142,153,159,160,161,162,163].

### 2.4. CPR/DPANN

The tree of life gives a primordial role to prokaryotes in phylogenetic evolution. The recent discovery of CPR (Candidate Phyla Radiation) and DPANN (acronym of the first five phyla, “Candidatus Diapherotrites”, “Candidatus Parvarchaeota”, “Candidatus Aenigmarchaeota”, Nanoarchaeota and “Candidatus Nanohaloarchaeota”) has generated new knowledge concerning the place of prokaryotes in the evolutionary processes of life. These entities have the general characteristics of prokaryotes but present original peculiarities (volume close to the theoretical minimal cell volume, i.e., 0.008 µm^3^, genome and reduced metabolic capacities consistent with a symbiotic lifestyle, and cellular components, e.g., ribosome, of unusual composition) which make them atypical prokaryotes and potentially the smallest known life form [4]. The evolutionary origins of the CPR and DPANN radiations in the two domains of bacteria and archaea, respectively, are still pending because the exact phylogenetic position of some of these entities in the tree of life is uncertain and controversial [4,164,165]. The hypothesis that some of these entities may have appeared during a dramatic but heterogeneous episode of genome reduction, or may have originated from a protogenote community and co-evolved with other prokaryotes, has recently emerged [165]. Considering the latter hypothesis and in a cell-centered view of life, CPR and DPANN could represent the smallest and simplest life form known from the last universal common ancestor (LUCA) and/or protogenotes [4,166,167]. These minimalist living entities could thus bridge the gap and establish the continuum between non-cellular but compartmentalized nano-entities (vesicles and viruses) and more complex cellular life forms.

**Figure 4:**
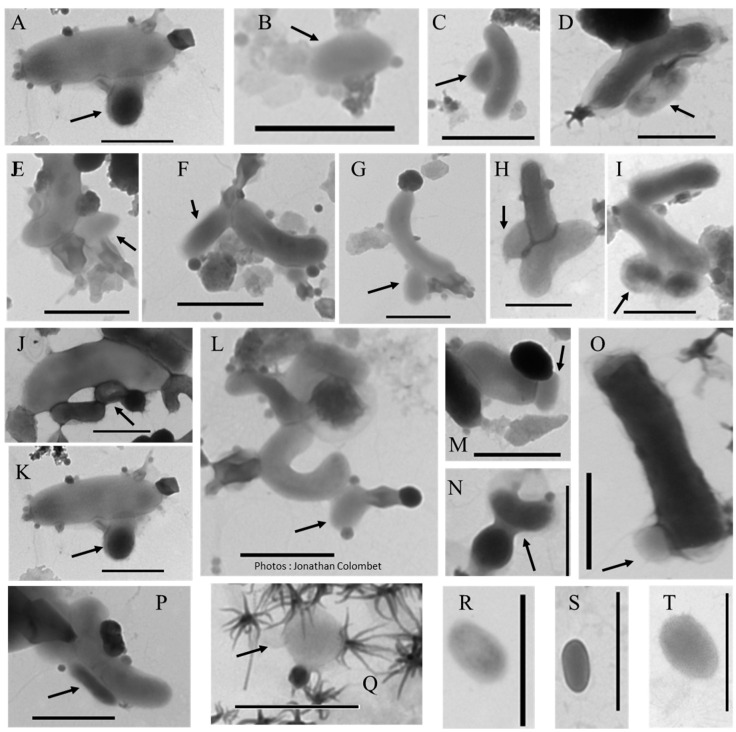
Transmission electron micrographs of attached (**A**–**Q**) or free (**R**–**T**) femtoplankton-like prokaryotes. Arrows indicate the target particles when the samples are heterogeneous. Scale bar: 500 nm.

Genome analyses and rare observations indicate that CPR and DPANN have the smallest genomes and cell size in the cellular world ([4] and references herein) (Figure 4). For example, the first member of Nanoarchaeota, *N. equitans*, is characterized by small cells, only 400 nm in diameter (volume = 0.0335 µm^3^), and codes for one of the smallest known archaeal genomes (0.49 Mb) [168,169]. Slightly larger genomes (0.64–1.08 Mb) of other ultra-small archaea (Parvarchaeota and Micrarchaeota) with cell volumes as low as 0.009 µm^3^, have since been discovered [170,171,172], as well as some nanosized Nanohaloarchaea (0.1–0.8 µm) [173,174,175,176]. Reduced genome and cell size are also characteristics of many groups of CPR bacteria. For example, a reduced genome of less than 0.694 Mb has been recorded for the candidate population OD1 [177], with a few ultra-small bacteria of the shortest length (less than 179 nm) and an assumed minimal volume close to 0.004 µm^3^ [178]. Most of the CPR is filterable onto 0.2 µm filter ([4] and references herein). Some CPR and DPANN species are characterized by sparse metabolisms, with limited catabolic and anabolic capacities, consistent with a symbiotic lifestyle ([4,164,165] and references herein). These authors pointed out that CPR and DPANN entities are not monolithic in terms of metabolism but rather harbor a diversity of metabolic capacities, consistent with a range of lifestyles ranging from obligatory symbionts or putative parasites to free-living mode, depending on their degrees of dependence on other organisms (prokaryotes or eukaryotes). The characteristics of CPR and DPANN call into question the fact that they are cellular life forms. Unlike vesicles and viruses, their ability to code genetic systems for cell division and to transform energy and carbon compounds, coupled with the existence of easily recognizable ribosomes (often of unusual composition), clearly distinguish them as cellular living organisms [4]. They therefore represent a substantial part of the diversity of bacteria and archaea domains. The CPR seems to be a monophyletic radiation with at least 74 phylum-level lineages while DPANN encompasses at least 10 different lineages [4,164]. In addition to terrestrial and animal microbiomes, these organisms were found in many aquatic environments, including acidic, alkaline, and hypersaline habitats, freshwater, and marine ecosystems ([4] and references herein).

Although these ubiquitous and diverse entities are recognized as the smallest known life form, the lack of an autonomous development in some of them opens a new path at the root of the tree of life to a group of organisms that are unable to reproduce by themselves. Finally, Lannes et al. [179] mentioned that CPR and DPANN superphyla may not be the only prokaryotes found in femtoplankton and they anticipated the discovery of new autotrophic aquatic nano-organisms with the development of single cell genomics.

### 2.5. Something New in the Femtoplankton

**Figure 5:**
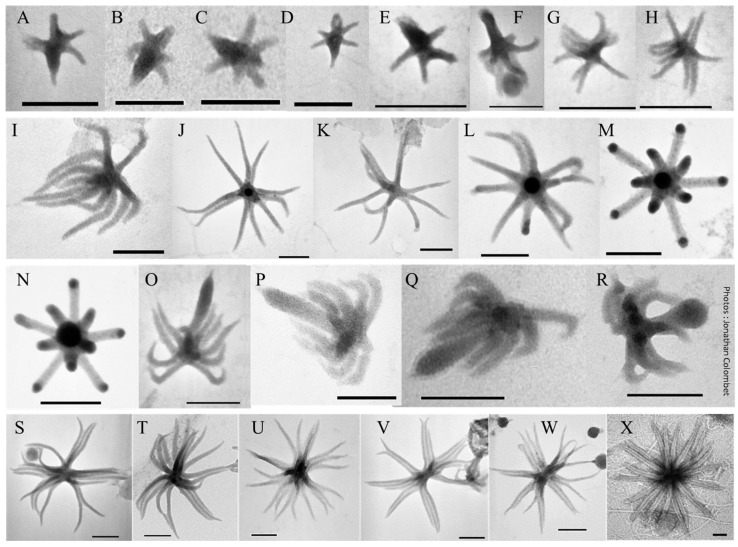
Transmission electron micrographs of different morphotypes of aster-like nanoparticles (ALNs). (**A**–**H**) 4–10-armed forms. (**I**–**M**) 11-armed forms and their budding 11-armed variants (**N**–**R**) with elongated and swollen bud-like excrescences. (**S**–**X**) 20-armed forms. Scale bars = 100 nm.

Over the past decade, the discovery of BMOPs, EVs and CPR/DPANN (see above) has significantly increased the complexity of the femtoplankton environmental fraction previously considered to be composed primarily of viruses [2]. This perception, discussed here, has been recently enriched by the discovery of mysterious aster-like nanoparticles (ALNs, Figure 5). These new femtoplankton particles, whose origin is unknown, do not belong to any previously defined environmental entities (see [5]). Selected-area electron diffraction of ALNs revealed an amorphous structure, mainly composed of carbon, oxygen, calcium and nitrogen. Trace amounts of potassium were also identified in association with the particles. ALNs are presumably formed of organic components [5]. ALNs are original pleomorphic nanoparticles (Figure 5) exhibiting puzzling aster-like shapes with arm-like outgrowths protruding from a central core. Three dominant morphotypes emerged based on the size and number of arms (4-, 11- and 20-armed forms). Some appeared endowed with a singular bud-like appendix that seemed to arise from the center of symmetry of the particle. The sagittal sections of the arms reveal a tubular appearance, with an area of electron light surrounded by a wall-like structure. Their average length ranges from 110 to over 439 nm, with volumetric estimates of less than 0.0014 µm^3^. Despite positive nucleic acid labelling, the presence of nucleic acids in ALNs remains to be proven [5]. The hypothesis of a support of heredity is supported by the occurrence of the same ALN morphotypes regardless of the environmental context and the recurrent radial symmetry of the particles, which might reflect a developmental relationship between the morphotypes [5]. We supplemented these unusual and original descriptive characteristics with development studies of ALNs in vitro and in situ. These include sensitivity to biocidal treatments, changes in ALN abundance in the absence of potential host cells, marked seasonal dynamics and developmental processes of ALNs that confirm their originality and question their origin [5]. We have also shown that ALNs are ubiquitous entities capable of maintaining themselves in most continental and coastal aquatic environments (lakes, rivers, marshes, estuarine area) [180]. The positive correlation between prokaryotic abundance and ALN recorded between all environments considered in this study, and the close physical contact between ALNs and prokaryotes displayed in [5], suggest a potential link between prokaryotes and ALNs. Future work is required to elucidate the origin, composition and ecology of these entities, until now unclassified, and their place in the evolution of life.

**Figure 6:**
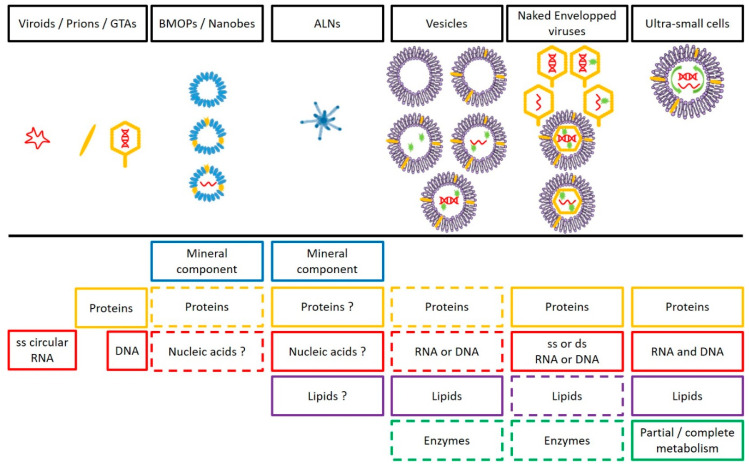
Schematic overview of the main components and of organizational complexity (increase in complexity from left to right) of the femtoplankton entities mentioned in this review. The question mark (?) represent uncertainty about the presence/absence of this compound in the target entity. The dotted line (--) means “Optional”. GTAs = gene transfer agents, BMOPs = Biomimetic mineral–organic particles, ALNs = aster-like nanoparticles. Note that the tail (mainly an attribute of bacteriophages) is not present in all naked viruses.

**Figure 7:**
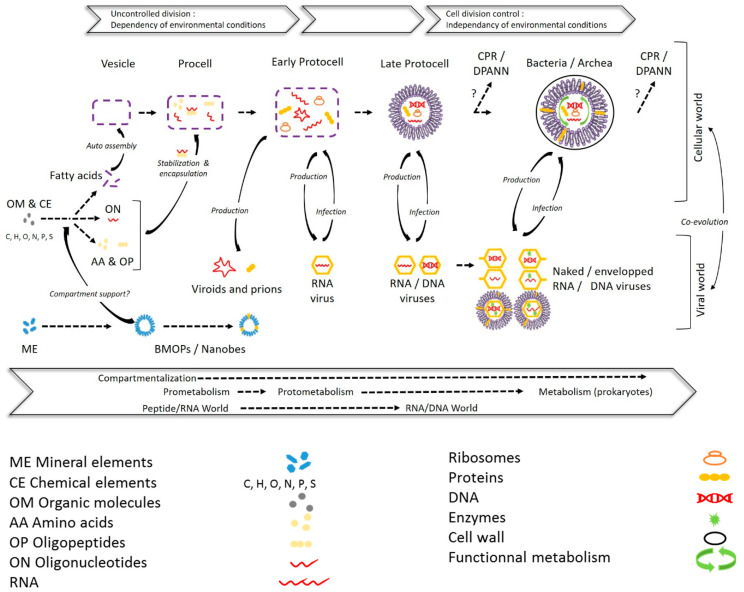
Significance of femtoplankton entities in prebiotic evolution: a potential pathway to the cellular and viral world. Early stages of evolution leading to a procell are based on the hypothesis of a prebiotic peptide/RNA world developed in [181]. In these early stages biomimetic mineral–organic particles (BMOPs)/nanobes, as a potential support (inside or fixed outside) of prebiotic organic chemistry, could have paved the way for the formation of organic molecules. The evolution of a procell toward the first protocell can be achieved after encapsulation and compartmentalization of the primitive elements into fatty acid vesicles prior to evolution by unregulated and error-prone way division, depending on environmental conditions [47,182]. Complexification of biochemical (metabolic) and replicative systems, as well as membrane/cell walls, during the protocell stages has led to divisions that are more independent of environmental conditions [182] and to the initiation of the cellular and viral world [94]. The emergence of Candidate Phyla Radiation/Diapherotrites Parvarchaeota Aenigmarchaeota Nanoarchaeota Nanohaloarchaeota (CPR/DPANN) from protocell or prokaryotic communities is adapted from [165].

Overall, the discovery of ALNs, following that of diverse and ubiquitous BMOPs/nanobes, EVs, and femtoplankton prokaryotes, suggests that femtoplankton could host novel types of other ultra-small particles that could provide new insights into biodiversity and the functioning of the aquatic environment. The main characteristics of the femtoplankton entities and their organizational complexity are summarized and schematized in Table 1 and Figure 6. This overlooked richness represents an unexpected windfall for understanding the evolutionary processes leading from minerals to the emergence of life on the earth (Figure 7). Indeed, femtoplankton entities can be placed in the context of prebiotic evolution by marking out the potential pathway to the cellular and viral world. In the early evolutionary stages, based on the hypothesis of a prebiotic peptide/RNA world developed in [181], BMOPs/nanobes, as potential supports (inside or fixed outside) of prebiotic organic chemistry, could have paved the way for the formation of primitive elements (organic molecules). The evolution of a procell towards the first protocell could have been achieved after encapsulation and compartmentalization of the primitive elements into fatty acids vesicles prior to evolution by unregulated and error-prone way division, depending on environmental conditions [47,182]. The complexification of the biochemical (metabolic) and replicative systems, as well as of the membrane/cell walls, during the following protocell stages would have led to divisions more independent of environmental conditions and to the initiation of the cellular and viral world [94,182]. CPR/DPANN emerged from late protocell stages or from prokaryotic communities as suggested in [165].

## 3. Quantitative and Functional Significances of Femtoplankton

One of the peculiarities of femtoplankton entities is their widespread distribution. The femtoplankton is present in all possible aquatic ecosystems. Viruses, vesicles and gene transfer agents as symbionts of prokaryotes are everywhere; they thrive from hot springs to polar glaciers, from acidic to alkaline environments, from freshwater to hypersaline systems ([48,50,51,93,121,122,123,124,125,126,127,128,159] and associated references). CPR/DPANN have also been listed in a wide variety of environments [4]. BMOPs/nanobes have been found in marine water and in some extreme environments [26,27,33,41]. ALNs, although data are still sparse, appear to be salinity-tolerant and colonize a wide variety of freshwater ecosystems [180]. Each environment has its own unique diversity of femtoplankton entities. Some types or species are endemic or specific to a given ecosystem under given conditions, others are more tolerant of variations in the environment and are widely distributed. The endemicity and transbiome invasion (e.g., marine-freshwater) of viruses and some femtoplankton prokaryotes are discussed in [183]. Movements or transfers from one ecosystem to another have been demonstrated for ALNs or viruses, for example, which can move along a watershed or through atmospheric systems [181,184,185]. Thus, femtoplankton entities are certainly the most diversified and widespread in the biological world. Capturing their diversity and specific abundance and comparisons between ecosystems is a challenge. By their diversity and composition, femtoplankton entities represent a huge reservoir of mineral and organic molecules. This reservoir makes them an essential player in the circulation, availability and transfer of elements affecting the biogeochemistry of their environment. The catabolic or anabolic metabolisms expressed in some of them or the potential symbiotic lifestyle in others, mean that these femtoplankton entities are not only an essential driving force in the diversification of aquatic organisms, but are also a significant driving force in the flow of matter and energy circulating in aquatic ecosystems. The following section reviews the quantitative and functional importance of the femtoplankton compartment according to their origin and composition as described above.

### 3.1. Quantitative Importance

Many efforts have been made to estimate the diversity of BMOPs/nanobes, EVs, GTAs and CPR/DPANN in the aquatic environment (see above). Nevertheless, estimates of their quantitative importance are still very rare. Although Wu et al. [33] mentioned that seawater contains a relatively high particle-seeding potential, to our knowledge, no data on the abundance of BMOPs or nanobes in the aquatic environment is available. Little more information is available for EVs. In a rare field study, Biller et al. [186] suggested that EV concentrations range from 10^5^ to 10^6^ vesicles per ml of sea water. To our knowledge, there are no data available about the abundance of GTAs in aquatic ecosystems. Genomic or proteomic studies of CPR and DPANN are increasingly documented, leading to a better consideration of their wide diversity [187,188,189]. However, to our knowledge there are no reports of their density in water neither as episymbionts (attached to a cell) nor as free-living elements. In a specific study, we reported significant amounts of ALNs in contrasted aquatic ecosystems [5]. These ubiquitous entities fluctuate spatially and temporally, with values ranging from undetectable to 9.0 ± 0.5 × 10^7^ particles·mL^−1^ [5]. As for other femtoplankton entities, the assessment of their quantitative importance requires a strong consideration in future work. Conversely, the quantitative importance of viruses has been widely documented and reviewed [93,122,123,190,191,192]. More than 10^30^ viruses can exist in aquatic environments at any given time [122]. Their abundances vary spatially and temporally up to estimates exceeding 10^8^ viruses·mL^−1^ [93,123,192]. Their current biomass has been estimated to be equivalent to 75 million blue whales (approximately 200 million tons of carbon) [193]. The abundance of RNA viruses can match or exceed that of DNA viruses [194]. Viruses are perhaps the most abundant biological entities on earth. The high abundances of EVs, CPR/DPANN and ALNs raise the question of the real quantitative contribution of viruses. Indeed, most estimates of viral abundance are based on counting of “virus-like particles” through positive nucleic acids labeling. These estimates probably lead to an overestimation of true viruses by counting all potential nucleic acid carriers described above, i.e., BMOPs/nanobes, EVs, GTAs, CPR/DPANN and ALNs [5,195]. The extent of this overestimation could have a fundamental impact on the ecological roles of viruses. Soler et al. [196] suggested that EVs could outnumber true viral particles in some aquatic environments and Colombet et al. [5] reported that ALNs can account for up to 40% of virioplankton counted by transmission electron microscopy.

There is an evident lack of data on the relative quantification of the recently discovered and overlooked femtoplankton components, both temporally and spatially. A combination of electronic microscopy and nucleic acid-based methods is needed to reveal the relative contribution of each of the femtoplankton categories [5]. In the future, such a consideration appears to be fundamental in deciphering the global importance of femtoplankton in the functioning of aquatic ecosystems and the related biogeochemical cycles.

### 3.2. Potential Ecological Importance

Estimating the overall functional importance of femtoplankton is a challenge for the future. This requires considering not only the diversity and quantity but also the biological/physiological state (composition, lifestyle, activity, etc.) of each of its representatives and the environmental contexts. A first approach is to speculate on the specific potential significance of each of the femtoplankton entities.

#### 3.2.1. BMOPs/Nanobes

These entities are overlooked in aquatic systems and very little data are available on their putative ecological importance. Like all femtoplankton entities, BMOP/nanobe biomass may play a crucial role in the circulation, availability and transfer of matter in the environment [33].

The assumed ecological significance of these entities could be inferred from biomedical sciences. Yaghobee et al. [32] reported several roles for some of these entities in calcification-related human diseases. Breitschwerdt et al. [197] and Barr et al. [34] reported their occurrence in terrestrial mammals. It is therefore very likely that the BMOPs/nanobes can play an important role in the health of marine animals, which by inference suggests an ecological role in the environment. çiftçioglu and Kajander [198] reported interactions (endocytosis) with cultured mammalian cells involving potential cytotoxicity. Such interactions with microbes in the environment could have a great implication for the receptor cell biology, although these are hypothetical and remain to be fully explored.

#### 3.2.2. Extracellular Vesicles

A little more information about the ecological significance of vesicles in aquatic systems is available. Gill et al. [48] reported that EVs, as carriers of various molecular cargoes from cell to cell, can modify cellular physiology (stress response, intercellular competition, pathogenicity and detoxification) and can play important roles in all types of intercellular interactions. They can be involved in the quorum sensing, acclimatization to nutrient limitation, morphological plasticity and trapping of toxins and antibiotics [51]. Additionally, EVs as carriers of genetic information between cells have been proposed as a novel vehicle for horizontal gene transfers (HGT), in addition to the well-known related mechanisms of transformation, transduction and conjugation [64,199]. As a result, they can significantly modify the gene pool and associated metabolic capacities of their receptors. Interactions between EVs and viruses have also been documented [48,200]. EVs have the potential to regulate host–virus dynamics [200]. Some EVs can propagate the viral genome or plasmids [91]. EVs can sometimes act as decoys to limit viral infection, while viruses can manipulate the production of EVs from infected cells to their own advantage [201,202]. As consequence, several ecological roles can be inferred for EVs, including their influence on ecology and community structure, the trophic-level interactions and their impact on the carbon cycle [51,52,53,54,55,56,57,58,59,60,61,62,63,64,65,66,67,68,69,70,71,72,73,74,75,76,77,78,79,80,81,82,83,84,85,86,87,88,89,90,91,92,93,94,95,96,97,98,99,100,101,102,103,104,105,106,107,108,109,110,111,112,113,114,115,116,117,118,119,120,121,122,123,124,125,126,127,128,129,130,131,132,133,134,135,136,137,138,139,140,141,142,143,144,145,146,147,148,149,150,151,152,153,154,155,156,157,158,159,160,161,162,163,164,165,166,167,168,169,170,171,172,173,174,175,176,177,178,179,180,181,182,183,184,185,186]. Nevertheless, as with BMOPs/nanobes, these potential roles are largely derived from biomedical sciences and remain to be extensively explored in natural environments.

#### 3.2.3. Viruses and Gene Transfer Agents

Microbial ecologists have devoted more effort into understanding the functional importance of viruses in aquatic environments [93,95,104,122,123,180,194,203,204,205,206,207,208,209]. These studies reported that viruses are major components of the aquatic food web, not only as parasites that can lead to cell death, but also as a powerful weapon able to manipulate the life histories, evolution and ecology of their hosts.

Suttle [122] reported that every second, approximately 10^23^ viral infections occur in the ocean. These infections are a major source of mortality, and cause disease in a wide range of organisms, from shrimp to whales. Through them, viruses contribute to both top-down and bottom-up control of the microbial community [207]. Viruses directly influence the abundance of aquatic communities. Lytic viruses may account for up to 50% of bacterial mortality in the pelagic ecosystems and can abruptly terminate eukaryote algae blooms [202,210,211,212,213]. Bossart and Duignan [95] noted that viral infections also have major effects on the health of marine mammals, including neoplasia, epizootics and zoonoses. As a major source of mortality, lytic viral infections considerably affect biogeochemical cycles. The fate of matter produced by lysis can follow a different pathway, from direct remineralization/regeneration by the microbial loop, which can support a higher microbial biomass, to export by aggregation and sedimentation [93,203,214,215]. Viral infection can alter cell stoichiometry and uptake rates [204,216]. Zimmerman et al. [208] discussed how metabolic reprogramming of host cells during lytic viral infection alters the nutrient cycle and ocean exports of carbon. They reported that viral infection transforms host metabolism through metabolic genes encoded by the virus, whose functions appear to alleviate energy and biosynthetic limitation in viral production. They emphasized the importance of the physiological state of the host cell and environmental conditions in the regulation of these processes.

Viruses are also important drivers of microbial diversity [93,217,218]. Two concepts can explain this power: the “antagonistic coevolution” (arms race) and “killing the winner”. In the concept of “antagonistic coevolution”, hosts and viruses coevolve in order to escape lethal infections for the hosts, a situation that can make surrounding hosts resistant to viruses [219]. In the “killing the winner” model, viral predation of temporarily abundant and specific hosts can weaken the between-host competition for resources and promote the coexistence of host diversity by allowing the growth of non-abundant or rare host species [217,220,221,222]. Viruses affect also the diversification and physiology of aquatic hosts through horizontal transfers of genetic materials [223,224]. One example is the transfer of photosynthesis genes between viruses and their hosts *Prochlorococcus* [225]. Ramisetty and Sudhakari [226] underlined that the temperate prophages are one of the most significant drivers of bacterial genome evolution and sites of biogenesis of genetic information. Nasir et al. [224] noted that phage conversion during transduction alters host physiology with respect to metabolism, pathogenicity, and niche adaptation. Although lacking metabolic activities, viruses can profoundly affect geochemical cycles by modelling the diversity and activity of their potential hosts.

GTAs are unusual vehicles for HGT, which appears to be an hybrid of bacteriophage transduction and natural transformation [153]. McDaniel, et al. [227] reported frequencies of antibiotic gene transfer by GTAs in in situ marine microcosms that were orders of magnitude greater than any other known mechanism. The transferred genes can enhance fitness or resilience and have the potential to drive bacterial evolution and genome plasticity, including the spread of virulence and antimicrobial resistance genes [228]. The ecological significance of GTAs is probably underestimated because they are difficult to distinguish from viral particles.

#### 3.2.4. CPR/DPANN and Other Femtoplankton Prokaryotes

The main ecological implications of the femtoplankton entities described above are related to their composition (BMOPs/nanobes, vesicles, viruses), their ability to transport and transfer various molecules and genetic material (EVs, viruses, GTAs) and/or their “parasitic” lifestyles (viruses) which can modulate the physiology and ecology of receptor cells (prokaryotes or eukaryotes). Until now, CPR/DPANN and other ultra-small prokaryotes are the only femtoplankton entities capable of metabolic activity. Although description of their potential metabolic activities is still in its infancy, this could profoundly impact biogeochemical cycles in the aquatic environment.

Castelle et al. [4,229] and Anantharaman et al. [230] have demonstrated that members of CPR and DPANN superphyla have genetic supports able of encoding molecules involved in numerous autotrophic or heterotrophic reactions. For example, some CPR and DPANN have Rubisco type II/III genes, while others have gene-encoding enzymes involved in the carbon, nitrogen, sulfur and hydrogen cycles [230,231]. Nevertheless, most CPR/DPANN lack parts of the central metabolic pathways, including nucleotides, amino acids and lipid biosynthesis and require a host to complete their life cycle [4,152,193,232]. Achievement of the metabolic potential of episymbiotic CPR/DPANN is therefore dependent on the presence and availability of their hosts. The corollary of these interactions is the potential impact on the activities and metabolic capacities of the organisms on which they depend [4]. On a larger scale, Anantharam et al. [230] revealed evidence of extensive interconnection between the metabolisms of coexisting community members. These interrelationships are likely necessary to complete many biogeochemical pathways. This does not exclude the notion that some CPR/DPANN seem to have the genetic potential to be free-living, with aerobic and/or fermentative heterotrophic behavior [4,232]. These discoveries are complemented by Lannes et al. [179] who have demonstrated that ultra-small marine prokaryotes, not necessarily CPR or DPANN, collectively harbor the genes required for the complex metabolism in carbon fixation, which could significantly increase their potential involvement in biogeochemical cycles. Lannes et al. [179] then anticipated that the discovery of new autotrophic marine nano-organisms with novel metabolic capacities is not impossible.

The potential ecological importance of CPR/DPANN, or other ultra-small prokaryotes is currently known through metagenomic and proteomic analyses or co-cultures of rare species. Therefore, their ability to express their genetic potential and to manipulate the metabolic potential of their hosts (symbiont, presumed new parasites) remains to be explored under contrasted environmental conditions, in order to estimate the overlooked ecological significance of these ultra-small prokaryotes.

#### 3.2.5. ALNs

The ecological role of the ALNs is currently unknown but could be potentially important. The total biomass of ALNs during bloom periods is likely to mobilize circulating mineral and organic nutrients to the detriment (competition?) of other microbial communities in aquatic ecosystems. For example, ALNs could be of great significance in the homeostasis of Ca in aquatic systems due to their high calcium composition. In addition, direct interplay with bacteria could significantly influence energy and matter flows mediated by prokaryote compartment [5]. The composition and activity of ALNs and interactions with the prokaryotic compartment remain to be confirmed and clarified to better understand the potential ecological role of ALNs. Clearly, these entities are new actors in the matter and energy flows circulating in aquatic systems which will have to be considered in future work.

**Figure 8:**
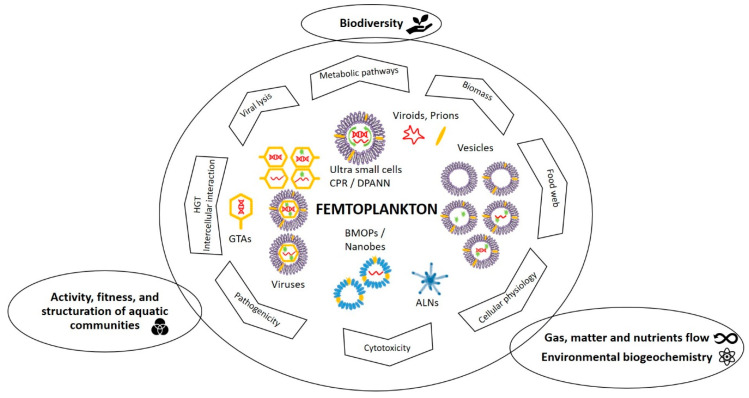
Schematic overview of the specific and major ecological roles of femtoplankton entities mentioned in this review. BMOPs = Biomimetic mineral–organic particles, ALNs = aster-like nanoparticles, CPR/DPANN = Candidate Phyla Radiation/Diapherotrites Parvarchaeota Aenigmarchaeota Nanoarchaeota Nanohaloarchaeota, GTAs = gene transfer agents, HGT = horizontal gene transfers.

Overall, recent evidence of numerous, diverse and ubiquitous, metabolically active (femtoplankton prokaryotes) or not (BMOPs/nanobes, EV), femtoentities as well as mysterious ALNs, implies a deep reconsideration of the diversity and ecological significance of femtoplankton. Historically considered through viral activity alone, this ecological significance may be greater than previously considered. Figure 8 reviewed the ecological potentials of femtoplankton representatives in the environment. The overlooked diversity and the associated biomass of all these entities necessarily have a deep impact on the circulation of conservative elements and the related biogeochemical cycling. Through the spectrum of their activities and potential hosts, femtoplankton entities have the ability to interact with all components of plankton. Femtoplankton maintains a privileged relationship with pico- and nanoplankton by not only being a parasite (e.g., viral lysis [122]), but also a food source (e.g., protozoan grazing [233]), a development factor (e.g., symbiosis with CPR/DPANN [4,230]) or evolution promotors (e.g., HGT via virus [223,224], EVs [64,199], GTAs [228]). Femtoplankton models both the phenotype and genotype of their interacting hosts and thus could significantly impact the biodiversity and ecological functions of all the components of plankton.

The autonomous realization of metabolic pathways by free-living femtoplankton entities (i.e., femtoplankton prokaryotes) could also significantly expand the ecological importance of femtoplankton in geochemical cycles.

Finally, taken together, femtoplankton entities represent a powerful engineering weapon able to deeply affect biodiversity and matter and energy flows circulating in aquatic environments. Nevertheless, most of the potential effects of femtoplankton remain to be explored. Their estimation needs to consider not only the diversity and biology of their representatives, but also their ability to interact with other biological elements and to express their activities in environmental contexts.

## 4. Conclusions

This review highlights that the femtoplankton compartment hosts a huge diversity of unidentified and overlooked entities at the frontiers of our knowledge. From an evolutionary point of view, this source of new diversity could be essential to a better understanding of the processes leading from the mineral–organic phase to a cell-like living entity. Femtoplankton could present all the stages presumably involved in the life initiation. Moreover, it could be crucial in many ecological processes. It represents a powerful engineering weapon capable of profoundly affecting biodiversity and matter and energy flows circulating in aquatic environments. Finally, this review highlights the need to deepen our knowledge of this still largely unknown compartment.

## Figures and Tables

**Table 1:** Comparison of morphologies, some main constitutional elements and development strategies of femtoplankton entities.

		Gene Transfert Agents	BMOPs/Nanobes	ALNs	Vesicles	Viruses	CPR/DPANN
**Shape**		Tailed phage like-particle (polyhedral)	Coccoid, amoeboid, filamentous, ovoid…	Aster-like	Circular	Helicoidal, polyhedral, spherical, bacilliform…	Coccoid, ovoid…
**Size (nm)**		30–80 (capsid)	20–1000	110–439	20–2000	17–1950	ND–400
**Main Composition**	**Dominant Mineral Component**	/	CaSO_4_, CaCO_3_, Al_2_O_3_, …	Ca (and others?)	/	/	/
	**Nucleic Acids**	DNA	Controversial	ND	Optional: RNA or DNA	RNA or DNA	RNA and DNA
	**Genome Size**	(4 to 14 Kbp)	/	/	Dependent on the producer cell	(0.859 to 2473 Kbp)	(0.49 to 1.08 Mbp)
	**Proteins**	+	Optional	ND	Optional	+	+
	**Lipids**	/	ND	ND	+	Optional (envelopped viruses)	+
**Surrounding Structure (Nature)**		Capsid (proteic)	+ (ND)	+ (ND)	Membrane	Capsid (proteic) Envelop (mainly lipidic)	Membrane/Cell wall/Glycocalyx
**Lifestyle**		Symbiosis	ND	ND	/	From symbiosis to parasitism	Symbiont/free
**Multiplication Strategy**		Lysis	Symmetrical fission	ND	Budding	Spectrum from lytic to lysogenic	Cell division

Viroids and prions composed only of RNA molecule and proteins respectively are not shown in the table. BMOPs = Biomimetic mineral–organic particles, ALNs = aster-like nanoparticles, CPR/DPANN = Candidate Phyla Radiation/Diapherotrites Parvarchaeota Aenigmarchaeota Nanoarchaeota Nanohaloarchaeota. ND = not determined. / = absence. + = presence.
